# Plasma concentrations of anti-inflammatory cytokine TGF-β are associated with hippocampal structure related to explicit memory performance in older adults

**DOI:** 10.1007/s00702-023-02638-1

**Published:** 2023-04-28

**Authors:** Matthias Raschick, Anni Richter, Larissa Fischer, Lea Knopf, Annika Schult, Renat Yakupov, Gusalija Behnisch, Karina Guttek, Emrah Düzel, Ildiko Rita Dunay, Constanze I. Seidenbecher, Burkhart Schraven, Dirk Reinhold, Björn H. Schott

**Affiliations:** 1 Leibniz Institute for Neurobiology, Brenneckestr. 6, 39118 Magdeburg, Germany; 2 Center for Intervention and Research on Adaptive and Maladaptive Brain Circuits Underlying Mental Health (C-I-R-C), Jena-Magdeburg-Halle, Germany; 3 German Center for Neurodegenerative Diseases (DZNE), Magdeburg, Germany; 4 Institute of Cognitive Neurology and Dementia Research, Medical Faculty, Otto-Von-Guericke-University Magdeburg, Magdeburg, Germany; 5 Institute of Molecular and Clinical Immunology, Medical Faculty, Otto-Von-Guericke-University Magdeburg, Magdeburg, Germany; 6 Center for Behavioral Brain Sciences (CBBS), Magdeburg, Germany; 7 Institute for Inflammation and Neurodegeneration, Medical Faculty, Otto-Von-Guericke-University Magdeburg, Magdeburg, Germany; 8 Health Campus Immunology, Infectiology and Inflammation (GC-I3), Medical Faculty, Otto-Von-Guericke-University Magdeburg, Magdeburg, Germany; 9 Center for Health and Medical Prevention (CHaMP), Otto-Von-Guericke-University Magdeburg, Magdeburg, Germany; 10 Department of Psychiatry and Psychotherapy, University Medical Center, Göttingen, Germany; 11 German Center for Neurodegenerative Diseases (DZNE), Göttingen, Germany

**Keywords:** TGF-β, hippocampus, hippocampal subfields, explicit memory, cognitive aging, inflammaging

## Abstract

Human cognitive abilities, and particularly hippocampus-dependent memory performance typically decline with increasing age. Immunosenescence, the age-related disintegration of the immune system, is increasingly coming into the focus of research as a considerable factor contributing to cognitive decline. In the present study, we investigated potential associations between plasma levels of pro- and anti-inflammatory cytokines and learning and memory performance as well as hippocampal anatomy in young and older adults. Plasma concentrations of the inflammation marker CRP as well as the pro-inflammatory cytokines IL-6 and TNF-α and the anti-inflammatory cytokine TGF-β_1_ were measured in 142 healthy adults (57 young, 24.47 ± 4.48 years; 85 older, 63.66 ± 7.32 years) who performed tests of explicit memory (Verbal Learning and Memory Test, VLMT; Wechsler Memory Scale, Logical Memory, WMS) with an additional delayed recall test after 24 h. Hippocampal volumetry and hippocampal subfield segmentation were performed using FreeSurfer, based on T1-weighted and high-resolution T2-weighted MR images. When investigating the relationship between memory performance, hippocampal structure, and plasma cytokine levels, we found that TGF-β_1_ concentrations were positively correlated with the volumes of the hippocampal CA4–dentate gyrus region in older adults. These volumes were in turn positively associated with better performance in the WMS, particularly in the delayed memory test. Our results support the notion that endogenous anti-inflammatory mechanisms may act as protective factors in neurocognitive aging.

## Introduction

Structural and functional alterations of the hippocampus-dependent memory system in aging are a well-replicated finding (Gorbach et al. 2017; Nyberg 2017), and there is mounting evidence for age-related immune dysregulation as a potential factor contributing to age-related cognitive decline and ultimately clinically relevant memory disorders (Brosseron et al. 2022). Normal aging, while not usually considered a disease (Rattan 2014), is accompanied by a low-grade pro-inflammatory state commonly termed "inflammaging" (Franceschi et al. 2000). Life-long exposure to an accumulating antigen load is thought to act as a chronic stressor on the immune system and to promote chronic, asymptomatic, and inflammatory activity (De Martinis et al. 2005). Aging cells frequently enter a state of senescence, which is characterized by a silenced cell cycle and a pro-inflammatory phenotype ("senescence-associated secretory phenotype", SASP). SASP is defined by senescence-induced increased secretion of pro-inflammatory molecules like cytokines or chemokines (Basisty et al. 2020) (see http://www.saspatlas.com/), which in turn induce senescence in neighboring cells (Nelson et al. 2012; Acosta et al. 2013). Furthermore, reduced adaptive immunity in old age is thought to further stimulate activity of the innate immune system as a compensatory mechanism (Fülöp et al. 2017). The resulting pro-inflammatory state increases the risk for age-related diseases like cancer or cardiovascular disease and for cognitive dysfunction (Tracy 2003; Akbaraly et al. 2013). Notably, some individuals show relatively preserved memory performance in old age (Nyberg and Pudas 2019), and research in over-centenarians suggests that balanced anti-inflammatory activity in addition to the age-typical pro-inflammatory phenotype may constitute a protective factor counteracting the negative effects of inflammaging (Franceschi 2007).

A prominent anti-inflammatory cytokine is the transforming growth factor β (TGF-β). Mammalian TGF-β protein exists in three different isoforms (TGF-β_1_, TGF-β_2_, and TGF-β_3_), with TGF-β_1_ being most abundant (Dobaczewski et al. 2011). TGF-β_1_ exerts considerable anti-inflammatory and neuroprotective effects (Preller et al. 2007; Harder et al. 2014), and there is evidence that TGF-β_1_ is further involved in plasticity processes related to learning and memory. For example, mice treated with TGF-β_1_ before being exposed to amyloid-β exhibit less synaptic loss and better-preserved memory (Diniz et al. 2017), and, in rats, TGF-β_1_ protects against amyloid-β-dependent neurodegeneration and release of pro-inflammatory cytokines (Shen et al. 2014; Chen et al. 2015). Additionally, TGF-β_1_ can induce the regrowth of damaged neurons after axonal injury (Abe et al. 1996). TGF-β_1_ knock-out mice express lower levels of the synaptic marker synaptophysin and the plasticity-related protein laminin as well as lower synaptic density in hippocampal neurons (Brionne et al. 2003). Inhibition of TGF-β_1_ signaling pathways in the mouse hippocampus leads to reduced expression of long-term potentiation (LTP) and impaired object recognition (Caraci et al. 2015), and further studies suggest that TGF-β_1_ is involved in the expression of the late phase of LTP (Caraci et al. 2015; Mikheeva et al. 2019; Nenov et al. 2019). Despite considerable evidence for a contribution of TGF-β_1_ to cellular mechanisms of learning and memory in rodents, little is known about a potential role of TGF-β_1_ in human neural plasticity and memory. There is, however, evidence for a protective role of TGF-β_1_ in neurodegenerative and neuroinflammatory diseases, particularly in Alzheimer's disease and multiple sclerosis (Martínez-Canabal 2015; Diniz et al. 2017).

Based on the well-documented role of TGF-β_1_ in rodent hippocampal plasticity and memory and its neuroprotective effects in human disease, we aimed to assess a potential relationship between TGF-β_1_ concentrations, hippocampal structure and learning and memory performance in healthy humans, with a focus on older adults. To control for concurrent pro-inflammatory activity, we additionally determined plasma levels of the inflammation marker C-reactive protein (CRP) and of the pro-inflammatory cytokines interleukin 6 (IL-6) and tumor necrosis factor α (TNF-α). As cognitive measures, we employed well-established neuropsychological tests of explicit memory (Verbal Learning and Memory Test, VLMT; Wechsler Memory Scale, WMS) and performed automated hippocampal segmentation and volumetry of hippocampal subfields (Quattrini et al. 2020) using magnetic resonance imaging (MRI) in a previously described cohort of young and older adults (Soch et al. 2021a; b). We hypothesized that higher TGF-β_1_ plasma concentrations would be associated with larger hippocampal volumes and better memory performance, particularly in older adults. Since hippocampal neurogenesis occurs primarily in the subgranular zone (SGZ) of the dentate gyrus (DG) and the functionally related CA4 region (Bond et al. 2021), we additionally conducted a focused analysis in those subregions. According to previous studies showing that the volumes of the input regions of the hippocampus (i.e., DG, CA4, and CA3 regions) are positively correlated with verbal memory (Mueller et al. 2011; Travis et al. 2014; Aslaksen et al. 2018; Kirchner et al. 2023), we further hypothesized that larger volumes of the CA4 region and DG would correlate with better memory performance in the VLMT and WMS.

## Materials and methods

### Study cohort

#### Recruitment

Participants were recruited via local press in Magdeburg, the online presence (www.lin-magdeburg.de) and social media of the Leibniz Institute for Neurobiology, as well as flyers in shopping centers and at Otto von Guericke University events. Individuals interested in participating provided information about potential contraindications for participation via telephone interview before they were invited to the study. All procedures were approved by the Ethics Committee of the Medical Faculty of the Otto von Guericke University Magdeburg and conducted according to their guidelines (ethics approval number 33/15). All subjects provided written informed consent to participate in accordance with the Declaration of Helsinki (World Medical Association Declaration of Helsinki: ethical principles for medical research involving human subjects 2013).

#### Participants

In the present study, we investigated participants from a previously described cohort consisting of neurologically and psychiatrically healthy young and older adults (Table 1) who underwent multimodal phenotyping, including neuropsychology, MRI, and investigation of blood-based biomarkers as part of the *Autonomy in Old Age* research alliance. A detailed characterization of the cohort and description of testing procedures has been reported previously (Soch et al. 2021a; b; Richter et al. 2023). All participants were right-handed according to self-report. The Mini-International Neuropsychiatric Interview (M.I.N.I.; Sheehan et al. 1998; German version by Ackenheil et al. 1999) was used to exclude present or past psychiatric disorders. Further contraindications for participation included alcohol or drug abuse, the use of neurological or psychiatric medication, and major psychosis (schizophrenia, bipolar disorder) in a first-degree relative. For the purpose of the present study, additional contraindications were chronic infectious, autoimmune or other inflammatory diseases (e.g., Crohn's disease, rheumatic diseases, Hashimoto's thyroiditis, or celiac disease), as well as regular use of immunomodulatory or anti-inflammatory medication. After excluding participants with missing data, a total of 142 participants (57 young, 85 older) were available for data analysis.

**Table 1:** Demographics, cytokine plasma concentrations, hippocampal volumes, and memory performance

	Older (≥ 50 years)	Young (18–35 years)	Age effects
Size of cohort	*n* = 85	*n* = 57	
Age distribution	63.66 ± 7.32	24.47 ± 4.48	
Sex distribution (f/m)	53/32	34/23	*X*^2^ = 0.11; *p* = 0.740
IL-6 [pg/ml]	1.50 ± 0.99	0.85 ± 0.64	*t*_139.8_ = 4.71; *p* < 0.001
TNF-α [pg/ml]	0.39 ± 0.15	0.27 ± 0.21	*t*_94.5_ = 3.70; *p* < 0.001
TGF-β_1_ [pg/ml]	2897.37 ± 1517.86	3300.14 ± 1806.62	*t*_140_ = 1.44; *p* = 0.154
CRP [ng/ml]	2622.00 ± 2202.47	2416.8 ± 3059.14	*t*_140_ = 0.47; *p* = 0.643
Whole HC			
Left	3210.00 ± 292.16	3400.27 ± 293.84	*t*_140_ = 3.80; *p* < 0.001
Right	3298.54 ± 316.87	3452.24 ± 303.19	*t*_140_ = 2.88; *p* = 0.005
DG			
Left	276.89 ± 32.19	300.27 ± 35.13	*t*_140_ = 4.09; *p* < 0.001
Right	289.82 ± 36.34	302.53 ± 36.89	*t*_140_ = 2.03; *p* = 0.044
CA4			
Left	236.20 ± 25.17	248.24 ± 26.89	*t*_140_ = 2.72; *p* = 0.007
Right	247.09 ± 28.71	250.04 ± 30.25	*t*_140_ = 0.59; *p* 0.558
VLMT			
Learning	52.35 ± 9.24	68.23 ± 4.78	*t*_132.7_ = 13.39; *p* < 0.001
24 h recall	8.89 ± 3.53	14.26 ± 1.23	*t*_111.7_ = 12.89; *p* < 0.001
WMS			
Immediate recall	25.58 ± 6.54	32.30 ± 6.98	*t*_140_ = 5.84; *p* < 0.001
24 h recall	21.74 ± 7.18	30.14 ± 7.81	*t*_140_ = 6.60; *p* < 0.001

Sex (female/male) and age as well as cytokine and CRP concentrations (mean ± standard deviation) are shown

*HC* hippocampus, *DG* dentate gyrus, *VLMT* Verbal Learning and Memory Test, *WMS* Wechsler Memory Scale (Logical Memory subscale)

### Cognitive testing

#### General procedure of testing

After signing consent and privacy forms, participants completed questionnaires on general health and MRI contraindications, followed by the M.I.N.I. (see above). Participants older than 50 years additionally underwent the Mini-Mental State Examination (MMSE) (Folstein et al. 1975) to rule out MCI or dementia. Afterward, all participants completed the Multivocabulary Intelligence Test (MWT-B) (Lehrl 2005). This was followed by the actual computer-based cognitive test battery, which included a German version of the VLMT (see 2.2.2) (Helmstaedter 2001) and a German auditory version of the WMS for logical memory (see 2.2.3) (Härting et al. 2000). For a comprehensive description of the test battery, see (Richter et al. 2023).

#### Verbal Learning and Memory Test (VLMT)

The VLMT includes two lists of 15 semantically unrelated words, a study list, and a distracter list (Helmstaedter 2001). The experiment was divided into a learning phase and a recall phase. In the learning phase, the words of the first list were presented consecutively visually. After the presentation of all words, participants were asked to write down each word they could remember. This procedure was repeated five times in succession. Next, a second list was presented once, followed by a written recall. This list served as a distractor list and was followed by the recall phase, in which the words from the first list were to be written down. Further recall phases followed after 30 min and after 24 h.

#### Wechsler Memory Scale (WMS): logical memory

The subscale *Logical Memory* of the WMS was implemented as a slightly modified, auditory version (Härting et al. 2000). The test persons listened to two short stories over headphones, which they were asked to write down immediately after listening. Recall tests took place 30 min and 24 h later. The recalled stories were rated by two independent experimenters according to an evaluation sheet with 25 items (i.e., details from the stories), and thus, a maximum of 25 points could be obtained per story and recall delay.

### Determination of TGF-β plasma levels

#### Sample collection, processing, and storage

Venous blood samples were collected from all participants by healthcare professionals after informed consent, including two plasma tubes with sodium citrate as anticoagulant. Platelet-poor citrate plasma was prepared using a standardized two-step separation method (Reinhold et al. 1997). Citrate plasma samples were stored as aliquots in Eppendorf tubes at – 80 °C until measurement. Whenever the interval between the two test days was 7 days or more, a blood sample was taken for both test days.

#### Enzyme-linked immunosorbent assay (ELISA)

Plasma concentrations of TGF-β_1_ were determined via enzyme-linked immunosorbent assay (ELISA). Additional ELISA measurements were carried out to determine plasma levels of CRP and the pro-inflammatory cytokines IL-6 and TNF-α. Commercially available Quantikine^®^ ELISA kits (R&D Systems Inc., Minneapolis, MN, USA) were used for this purpose. For IL-6 and TNF-α, the high-sensitivity variant was used, as low plasma concentrations of these cytokines were expected in healthy individuals, and the high-sensitivity variant is characterized by a lower minimum detection dose.

### Magnetic resonance imaging

#### MRI data acquisition

MRI data were collected using two MRI systems (3 Tesla Verio-Syngo MR system, Siemens Medical Systems, Erlangen, Germany and 3 Tesla Skyra Fit MR system). For volumetric analyses, a T1-weighted 3D Magnetization Prepared Rapid Acquisition Gradient Echo (MPRAGE) image was acquired (TR = 2.5 s, TE = 4.37 ms, flip angle = 7°, 192 sagittal slices, in-plane resolution = 256 × 256, and isotropic voxel size = 1 mm^3^). In addition, high-resolution coronal T2-weighted images were acquired using a protocol optimized for medial temporal lobe volumetric analyses (TR = 3.5 s, TE = 353 ms, 64 coronal slices orthogonal to the hippocampal axis, in-plane resolution = 384 × 384, and voxel size = 0.5 × 0.5 × 1.5 mm^3^) (Richter et al. 2023).

#### Volumetric measurement of hippocampal subfields

Automated volumetric analysis of the individual participants’ hippocampi and their subfields was performed using FreeSurfer 6.0 (Fischl 2012) and the module for segmentation of hippocampal subfields, which is based on both *in* vivo scans of human participants and ex vivo scans of hippocampi from autopsy specimens (Iglesias et al. 2015). Previous analyses have confirmed the robustness of this protocol across different MRI scanners (Quattrini et al. 2020). In addition to the T1-weighted MPRAGE images, high-resolution coronal T2-weighted images (see 2.4.1) were used to improve segmentation accuracy (Dounavi et al. 2020).

### Statistical analysis

Statistical analysis was performed using Matlab R2018b (Mathworks, Natick, MA), SPSS Statistics v23 (IBM, Armonk, NY), and R, version 4.0.4 (https://www.r-project.org/), with RStudio version 1.4.1103 (RStudio Team 2021), employing the R packages psych (Revelle 2022) and sjPlot (Lüdecke 2022).

Main effects of age group and gender on cytokine concentrations, hippocampal volumes, and memory performance were assessed using MANOVAs followed by post hoc two-sample *t* tests. Whenever Levene’s test was significant, t tests for unequal variances (i.e., Welch’s tests) were employed.

To investigate a potential direct association of pro- and anti-inflammatory cytokines with learning and memory performance in the VLMT and in the WMS Logical Memory subscale, multiple linear regressions were computed with the concentrations of TGF-β_1_, CRP, IL-6, and TNF-α as well as age and gender as independent variables. An additional independent variable was the infection history of the subjects (i.e., self-report of "cold, flu, urinary tract infection or similar" or vaccination) within 4 weeks prior to testing (hence referred to as "immune event history"). The dependent variables were learning and memory performance and delayed recall in the VLMT and the WMS. The measure of learning performance for the VLMT was the sum of remembered items from learning sessions 1–5. For the WMS, the sum of the remembered items from both stories in the learning session served as measure of learning performance. Delayed recall performance was quantified as the number of recalled words or story items in the 24-h recall of the VLMT and WMS, respectively.

Statistical analysis of the relationship between TGF-β plasma concentrations and MRI data (i.e., hippocampal volumes and individual hippocampal subfields) was performed analogously to the behavioral data analysis. Multiple linear regressions were calculated with the concentrations of TGF-β, IL-6, TNF-α, and CRP as well as age, sex, and immune event history as independent variables and the volumes of the hippocampus or hippocampal subregions (DG, CA) as dependent variables, separately for each hemisphere. A correction for the false discovery rate (FDR) was applied for the number of regression analyses (*N* = 6). To assess for a potential association between hippocampal structure and memory performance, Pearson’s correlations were computed between the volumes of subregions that showed a robust association with cytokine plasma concentrations (*p* < 0.05, FDR-corrected) and performance in the VLMT (learning and delayed recall) and in the WMS (immediate and delayed recall).

## Results

### Age and gender differences in cytokine levels, hippocampal volumes, and memory performance

Table 1 displays the demographic data, cytokine levels, hippocampal volumes, and performance in the VLMT and WMS, separately for the two age groups. A MANOVA with age and gender as fixed factors and TGF-β_1_, IL-6, TNF-α, and CRP as dependent variables revealed significant effects of age group (Wilks’ *Λ* = 0.789; *F*_4,135_ = 9.00; *p* < 0.001) and gender (Wilks’ *Λ* = 0.919; *F*_4,135_ = 2.97; *p* = 0.022) as well as an age-by-gender interaction (Wilks’ *Λ* = 0.901; *F*_4,135_ = 3.67; *p* = 0.007). Post hoc two-sample *t* tests revealed significantly higher levels of IL-6, and TNF-α in older compared to young adults, but no age differences in TGF-β_1_, or CRP plasma concentrations (Table 1). Significant gender-related differences were observed for CRP in the younger cohort only, with higher levels in women (young: *t*_37.6_ = 4.17; *p* < 0.001; older: *t*_83_ = 0.46; *p* = 0.636). No gender differences in either age group were found for TGF-β_1_, IL-6, or TNF-α plasma concentrations (all *p* > 0.061).

Volumes of the hippocampus and of CA4 and DG subregions showed highly significant differences as a function of both age group (Wilks’ *Λ* = 0.618; *F*_6,133_ = 13.70; *p* < 0.001) and gender (Wilks’ *Λ* = 0.880; *F*_6,133_ = 3.04; *p* = 0.008) as well as a significant age-by-gender interaction (Wilks’ *Λ* = 0.905; *F*_6,133_ = 2.33; *p* = 0.036). Table 1 displays the results of the post hoc *t* tests, which showed that older adults had significantly lower volumes of the whole hippocampus bilaterally as well as of all subregions tested except for the right CA4 region. Regarding gender effects, post hoc *t* tests in older adults revealed significantly larger volumes of the whole hippocampus and of the subfields in men compared to women (all *p* < 0.043). In young adults, the same tendency was observed, but largely only at a trend level (0.019 < *p* (one-tailed) < 0.056). This is in line with the previous studies showing larger hippocampal volumes in males, which can be attributed to larger intracranial or total brain volumes (Tan et al. 2016).

As expected, memory performance was lower in older compared to young adults. A MANOVA with VLMT learning performance and 24 h delayed recall performance as dependent variables revealed a highly significant effect of age group (Wilks’ *Λ* = 0.459; *F*_2,137_ = 80.74; *p* < 0.001) but no effect of gender and no interaction (all *p* > 0.120). Post hoc two-sample *t* tests showed significantly higher performance in young compared to older adults in both learning and 24 h delayed recall (Table 1). Similarly, WMS logical memory performance was significantly lower in older compared to young adults (main effect of age group: Wilks’ *Λ* = 0.774; *F*_2,137_ = 20.02; *p* < 0.001; see Table 1 for post hoc two-sample *t* tests), but no effect of gender and no interaction (all *p* > 0.307).

### Association of TGF-β plasma concentrations with hippocampal structure

As TGF-β has previously been described to affect hippocampal neurogenesis, which occurs primarily in the SGZ, we hypothesized the subregions of the hippocampus most likely to be related to TGF-β_1_ plasma concentrations would be the DG and the CA4 region. To investigate potential associations between plasma concentrations of TGF-β_1_ or other cytokines (IL-6 and TNF-α) and hippocampal structures, we computed multiple linear regressions with the volumes of the entire hippocampus and the DG and CA4 regions as the dependent variable, separately for each hemisphere. Immune event history, age, and gender were included as additional independent variables.

**Fig. 1:**
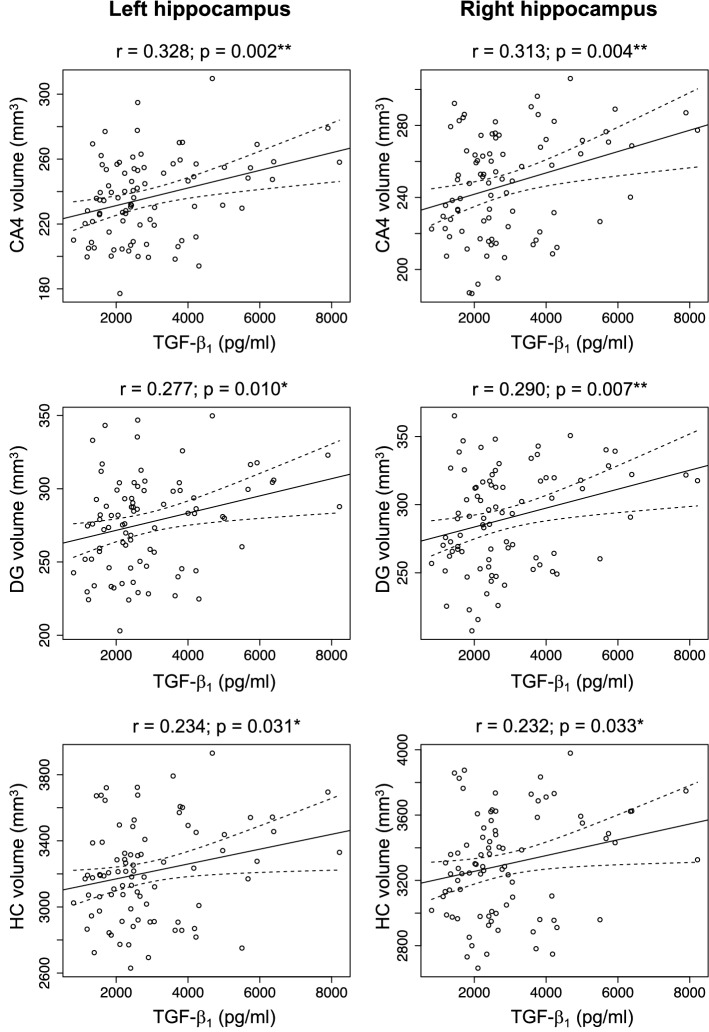
Correlation of TGF-β_1_ plasma concentrations with hippocampal subfield volumes. Pearson’s correlations are shown for the dentate gyrus (DG), CA4, and whole hippocampus, separated by hemisphere. *p < 0.05, two-tailed; **p < 0.01, two-tailed

TGF-β_1_ plasma concentrations were significantly positively correlated with the volumes of the CA4 and DG subregions as well as the volumes of the entire hippocampus in both hemispheres (all *p* < 0.050, FDR-corrected) in older adults. Other factors influencing the volumes of these regions in older adults were age and gender (all *p* < 0.032). Table 2 displays the adjusted regression coefficients and significance levels for all immune markers and anatomical regions, and Fig. 1 depicts the correlations between TGF-β_1_ plasma concentrations and hippocampal volumes. However, no effects of TGF-β1 plasma concentrations, age, or gender could be observed in young adults (all *p* > 0.060, uncorrected), suggesting that the positive relationship between TGF-β_1_ plasma levels and the hippocampal structure was restricted to older adults.

**Table 2:** Immune markers and hippocampal subfield volumes

	Whole HC		CA4		DG	
	Left	Right	Left	Right	Left	Right
TGF-β_1_						
std. β	***0.43***	***0.22***	***0.32***	***0.32***	***0.27***	***0.29***
CI	***[0.11 to 0.76]***	***[0.03 to 0.40]***	***[0.13 to 0.51]***	***[0.13 to 0.50]***	***[0.07 to 0.46]***	***[0.10 to 0.48]***
P	0.025**	0.023**	0.001**	0.001**	0.008**	0.003**
IL-6						
std. β	0.06	0.08	0.13	0.18	0.16	0.18
CI	[– 0.16 0.29]	[– 0.13 0.29]	[– 0.09 to 0.35]	[– 0.04 to 0.40]	[– 0.06 to 0.39]	[– 0.04 to 0.40]
P	0.566	0.465	0.242	0.099	0.154	0.105
TNF-α						
std. β	0.00	– 0.11	– 0.00	– 0.08	– 0.02	– 0.07
CI	[– 0.21 to 0.21]	[– 0.32 to 0.09]	[– 0.21 to 0.21]	[– 0.28 to 0.13]	[– 0.24 to 0.19]	[– 0.28 to 0.14]
P	0.999	0.285	0.994	0.466	0.833	0.505
CRP						
std. β	0.21	***0.22***	0.07	– 0.02	0.06	– 0.03
CI	[– 0.01 to 0.43]	***[0.01 to 0.44]***	[– 0.15 to 0.29]	[– 0.24 to 0.19]	[– 0.17 to 0.28]	[– 0.25 to 0.19]
P	0.066	0.039*	0.504	0.843	0.340	0.793
Immune event						
std. β	– 0.47	– 0.47	– 0.36	*** – 0.56***	– 0.26	– 0.47
CI	[– 0.99 to 0.06]	[– 0.97 to 0.03]	[– 0.88 to 0.15]	***[– 1.07 to – 0.06]***	[– 0.78 to 0.27]	[– 0.98 to 0.04]
P	0.080	0.063	0.160	0.029*	0.340	0.072

Results of multiple regression analyses are shown. Significant regression coefficients are depicted in bold font.

*std. β* standardized regression coefficients, *CI* confidence interval

*p < 0.05 uncorrected

**p < 0.05 FDR-corrected

### Correlations of hippocampal subfield volumes with memory performance

**Fig. 2:**
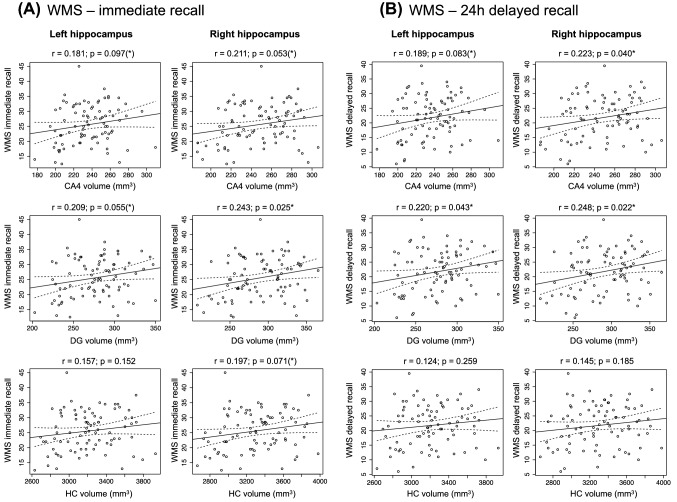
Correlation of hippocampal subfield volumes with memory performance (WMS Logical Memory). Pearson’s correlations are shown for the dentate gyrus (DG), CA4, and whole hippocampus, separated by hemisphere. **A** WMS immediate recall. **B** WMS 24 h delayed recall. *p < 0.05, two-tailed; (*)*p* < 0.05, one-tailed

To assess a potential relationship between the volumes of the hippocampus with memory performance in regions associated with immune markers, Pearson’s correlations between volumes and memory performance in the VLMT and WMS were computed for all regions showing a robust relationship (FDR-corrected *p* < 0.05) with at least one immune marker. As no robust associations were found in young adults, these analyses were restricted to the older participants in our cohort. We observed a positive correlation of the WMS Logical Memory scale with the volume of the right DG (*p* = 0.025, two-tailed) and, as a trend, also of left DG and the CA4 region bilaterally as well as the volume of the whole right hippocampus (all *p* < 0.05, one-tailed; see Fig. 2). Moreover, significant positive correlations with memory performance in 24-h delayed recall test of the WMS were observed for the volumes of the dentate gyrus bilaterally and the right CA4 region (all *p* < 0.05, two-tailed) and, as a trend, also for the left CA4 region (*p* = 0.042, two-tailed). On the other hand, for the VLMT, no significant correlations with the volumes of any of the hippocampal regions investigated were detected (all *p* > 0.096).

### TGF-β plasma concentrations and memory performance

In both the learning and the 24 h delayed recall phases of the VLMT, regression analysis confirmed worse performance of older adults compared to young adults (linear regression, complete sample, effect of age; learning: standardized *β* = – 0.70, confidence interval [CI] = [– 0.83 to – 0.57], *p* < 0.001; delayed recall: standardized *β* = – 0.66, confidence interval [CI] = [– 0.80 to – 0.53], *p* < 0.001). Within the group of older adults, linear regression analysis revealed significant effects of age and gender on learning performance (age: standardized *β* = – 0.34, CI = [– 0.56 to – 0.13], *p* = 0.002; gender: standardized *β* = 0.44, CI = [0.02–0.86], *p* = 0.038) and delayed recall (age: standardized *β* = – 0.31, CI = [– 0.53 to – 0.09], *p* = 0.006; gender: standardized *β* = 0.47, CI = [0.05–0.89], *p* = 0.028). We found, however, no effect of TGF-β_1_ plasma concentrations on learning or delayed recall performance (learning: standardized *β* = – 0.00, CI = [– 0.35 to 0.06], *p* = 0.157; delayed recall: standardized *β* = 0.00, CI = [– 0.30 to 0.10], *p* = 0.328). There were also no effects of plasma levels of other cytokines or CRP nor of immune event history on either learning or delayed recall performance in the VLMT (all *p* > 0.217).

In the WMS, regression also confirmed worse performance in older versus young adults in both immediate and 24 h delayed recall (complete sample, effect of age; immediate recall: standardized *β* = – 0.46, confidence interval [CI] = [– 0.63 to – 0.30], *p* < 0.001; delayed recall: standardized *β* = – 0.48, confidence interval [CI] = [– 0.64 to – 0.31], *p* < 0.001). Among older adults, we also found significant effects of age (immediate recall: standardized *β* = – 0.38, CI = [– 0.60 to – 0.17], *p* < 0.001; delayed recall: standardized *β* = – 0.28, CI = [– 0.51 to – 0.06], *p* = 0.014). As with the VLMT, however, no effect of TGF-β_1_ plasma concentrations on memory performance was found (immediate recall: standardized *β* = – 0.00, CI = [– 0.35 to 0.06], *p* = 0.157; delayed recall: standardized *β* = 0.00, CI = [– 0.30 to 0.10], *p* = 0.328). There were also no significant effects of plasma levels of other cytokines or CRP or of immune event history on either immediate or delayed recall performance in the WMS, nor an effect of gender (all *p* > 0.165).

## Discussion

Our results suggest that plasma concentrations of the anti-inflammatory cytokine TGF-β_1_ are associated with hippocampal CA4 and dentate gyrus volumes in older adults. The volumes of these hippocampal regions were in turn associated with immediate and particularly delayed recall performance in the WMS Logical Memory scale in the older participants.

### A potentially protective role for TGF-β_1_ in the aging hippocampus

While young and older participants did not per se show age-related differences in TGF-β_1_ plasma concentrations (see Table 1), specifically in older adults, higher concentrations of TGF-β_1_ were associated with hippocampal structure and particularly the volumes of the hippocampal CA4 and DG subregions. Previous rodent studies and investigations in human neurodevelopmental disorders have pointed to a role for TGF-β signaling in hippocampal development and maintenance (Stegeman et al. 2013; Oishi et al. 2016; Johnson et al. 2020). In adult mice, TGF-β can promote hippocampal LTP, particularly the transition from early to late-phase LTP (Caraci et al. 2015; Nenov et al. 2019), as well as adult neurogenesis in the DG (Gradari et al. 2021). Given the age-dependence of the association between TGF-β_1_ plasma concentrations and hippocampal structure in the present study, one possibility could be that effects of impaired TGF-β signaling during development may be compensated for during young adulthood, but, during aging, the capacity for compensation decreases. On the other hand, it must be noted that TGF-β_1_ plasma concentrations were measured at or around the time of testing in our study, and we cannot make statements about long-term availability of TGF-β_1_.

Therefore, in our view, a more likely or perhaps additional explanation for the age-dependence of the observed association could be related to the prominent role of TGF-β_1_ signaling in CNS, but also systemic inflammation. It has been recognized for over 2 decades that microglia and astrocytes in particular play a central role as mediators between peripheral, low-grade inflammation and the cellular mechanisms of learning and memory (Yirmiya and Goshen 2011). They also express increased pro- and anti-inflammatory cytokines in aging and respond to peripheral inflammatory processes (Sierra et al. 2007). For example, microglia secrete the anti-inflammatory cytokine IL-10 in response to an inflammatory stimulus, which in turn induces astrocytes to produce TGF-β, resulting in an inhibition of the microglial inflammatory response (Norden et al. 2014). However, aging astrocytes frequently show a blunted response to IL-10, resulting in lower levels of TGF-β_1_ expression (Norden et al. 2016) and, consequently to a shift toward a pro-inflammatory state in microglia (Diniz et al. 2017), which has a negative impact on neural plasticity (Kempermann and Neumann 2003; Yirmiya and Goshen 2011). While this could in principle explain a relationship between higher TGF-β_1_ plasma levels and larger volumes of the hippocampal CA4 region and DG, an explanation primarily based on neurogenesis may be questionable, as recent investigations cast doubt on the robustness of findings on adult neurogenesis in humans (Sorrells et al. 2021). Nevertheless, the role of chronic inflammation in synaptic dysfunction and loss and ultimately neurodegeneration is a well-documented finding (Yirmiya and Goshen 2011; Daulatzai 2014), and increased TGF-β_1_ levels have been found in studies of pharmacological neuroprotection (Hosseini et al. 2018; Wiciński et al. 2018). Furthermore, TGF-β_1_ also promotes the outgrowth of dendrites (Battista et al. 2006; Mathieu et al. 2010; Luo et al. 2016).

One limitation of the present study is that we could measure TGF-β_1_ concentrations only in plasma but not, for example, in cerebrospinal fluid (CSF). There is surprisingly little available literature on the relationship between CSF and plasma concentrations of TGF-β_1_. Previous studies that included measures from both CSF and blood have often treated those measures separately and not reported the correlations between them (Iłzecka et al. 2002; Liu et al. 2022). One study in patients with Alzheimer’s disease explicitly mentioned an absence of such correlations (Motta et al. 2007), but in that study, peripheral blood levels of TGF-β_1_ were measured in serum and were thus most likely largely attributable to TGF-β_1_ released from degranulated platelets (Grainger et al. 2000; Iłzecka et al. 2002). In the present study, we used a protocol that avoids contamination from platelet-derived TGF-β_1_ (Reinhold et al. 1997), but a potential relationship with CSF TGF-β_1_ remains nevertheless elusive. Furthermore, it must be noted that TGF-β_1_ is not able to penetrate the intact blood–brain barrier (BBB) (Kastin et al. 2003). On the other hand, BBB integrity declines with age, even in healthy individuals. The hippocampus and in particular the CA1 region, but also the DG seem to be particularly affected by age-related BBB permeability, while the BBB remains comparably intact in neocortical areas (Montagne et al. 2015). Furthermore, increased BBB permeability in aging elicits increased activation of TGF-β signaling pathways and increased expression of TGF-β_1_ in the hippocampus (Senatorov et al. 2019). Additionally, TGF-β_1_ has been implicated in angiogenesis and BBB assembly (Diniz et al. 2017), and might increase reactively in response to BBB damage.

### Hippocampal subregion structural integrity and memory performance in old age

While we observed no direct effect TGF-β_1_ plasma concentrations on memory performance, we found that the volumes of the hippocampal regions shown to be associated with TGF-β_1_ levels correlated with immediate and particularly delayed recall performance in the WMS Logical Memory scale in the older participants. Previous volumetric segmentation of hippocampal subregions and their association with mnestic abilities have revealed that the volumes of the input regions of the hippocampus (i.e., the DG and the CA4 and CA3 regions) are positively correlated with learning performance in well-established memory tests (Mueller et al. 2011; Travis et al. 2014; Aslaksen et al. 2018). On the other hand, the volumes of hippocampal output regions (i.e., CA1 and subiculum) are more related to delayed memory retrieval (Mueller et al. 2011; Aslaksen et al. 2018). This distinction is further supported by a functional MRI (fMRI) study on memory-related activations of hippocampal subregions (Eldridge et al. 2005). Nevertheless, caution is warranted, as other works have also yielded relationships between CA1 volume and learning performance as well as DG and CA4 volumes with performance in delayed retrieval (Aslaksen et al. 2018). Along the same line, a high-resolution fMRI study of successful encoding of information showed that encoding success correlated with activity in the CA1 region and subiculum, while activity in the DG, CA2, and CA3 was related to stimulus novelty (Maass et al. 2014). Evidence from rodent studies suggests that the volume of the CA4 region is associated with better spatial memory (Schwegler et al. 1990; Crusio and Schwegler 2005). To interpret these findings, one should keep in mind that “CA4” likely constitutes a misnomer, as, unlike CA1, CA3, and the small C2 region, CA4 mainly contains non-pyramidal cells and is functionally closely related to the adjacent DG (Amaral 1978; Amaral et al. 2007). As such, the region labeled “CA4” in the FreeSurfer-based segmentation of the hippocampus also contains the mossy fibers that originate from neurons of the DG, which was itself also associated with better memory performance in the older group, compatible with findings from previous studies (Zheng et al. 2018; Broadhouse et al. 2019; Kern et al. 2021; Wan et al. 2020). A larger volume in these regions might, for example, reflect both from more neurons in the DG due to better-preserved structural integrity and possibly neurogenesis as well as from a larger number of dendrites, possibly due to the role of TGF-β_1_ in promoting the outgrowth of dendrites (Battista et al. 2006; Mathieu et al. 2010; Luo et al. 2016).

### Clinical implications and directions for future research

Our results suggest that plasma TGF-β_1_ levels are associated with a better-preserved hippocampal structure in older adults, which in turn is associated with greater episodic memory performance. Notably, our current data are based on physiological variability of TGF-β_1_ plasma levels, and potential effects of raising the concentrations to supra-physiological levels, for example, by pharmacological intervention, warrant further investigation. Increased TGF-β_1_ plasma levels have, for example, been found in patients with multiple sclerosis and are further increased by immunomodulatory treatment with interferon- β1b (Nicoletti et al. 1998), suggesting that the treatment might augment an already active endogenous anti-inflammatory mechanism. Little is known, however, about potential cognitive effects of elevated TGF-β_1_ levels. Notably, CSF TGF-β_1_ concentrations are increased in Alzheimer’s disease (Swardfager et al. 2010), but their relationship with cognitive performance depends on the disease stage, with both unaffected and severely affected individuals showing a positive correlation with cognitive performance (i.e., MMSE), whereas mildly-to-moderately affected patients exhibit a negative relationship (Motta et al. 2007). Future research should further elucidate the relationship between TGF-β_1_ levels and brain, particularly hippocampal, integrity in clinical populations to clarify, for example, its role in inflammatory processes related to neurodegeneration in Alzheimer’s disease (Brosseron et al. 2022).

Beyond TGF-β, our results provide further evidence for a relationship between peripheral markers of inflammation and brain structure, particularly hippocampal structure, as well as neurocognitive functioning (Marsland et al. 2008; Yirmiya and Goshen 2011; Baune et al. 2012; Brosseron et al. 2023). Future studies should expand those works, for example, by employing functional MRI and derived biomarkers (Soch et al. 2021a; Richter et al. 2023). Another important direction for future research concerns gender differences in inflammaging. In the present study, we observed higher CRP levels in young, but not older, women compared to men, which have been reported previously by other groups and partly attributed to gender differences in body fat or estrogen levels (Khera et al. 2005, 2009; Lakoski et al. 2006). As we found no gender differences in older adults, future studies should explore the possibility that, for example, hormonal differences and their lifespan-associated changes might contribute to immunosenescence and associated neurocognitive alterations.

## Conclusions

Our results suggest that TGF-β_1_ plasma levels are associated with better structural preservation of the hippocampus in older adults, which is predictive for better episodic memory performance. The present data highlight the role of anti-inflammatory mechanisms as potential protective factors in neurocognitive aging.

## Data Availability

Access to de-identified raw data and R scripts used for data analysis will be provided by the authors upon reasonable request.
